# Effects of Physical Exercise on Neurofilament Light Chain and Glial Fibrillary Acidic Protein Level in Patients with Multiple Sclerosis: A Systematic Review and Bayesian Network Meta-Analysis

**DOI:** 10.3390/jcm14030839

**Published:** 2025-01-27

**Authors:** Aitor Blázquez-Fernández, Víctor Navarro-López, Selena Marcos-Antón, Roberto Cano-de-la-Cuerda

**Affiliations:** 1 International PhD School, Rey Juan Carlos University, 28008 Madrid, Spain; aitorblazquezfernandez@outlook.es; 2 Asociación de Leganés de Esclerosis Múltiple (ALEM), Leganés, 28915 Madrid, Spain; 3 Department of Physical Therapy, Occupational Therapy, Physical Medicine and Rehabilitation, Faculty of Health Sciences, Rey Juan Carlos University, Alcorcón, 28922 Madrid, Spain; roberto.cano@urjc.es; 4 Department of Health Sciences, Universidad Villanueva, 28034 Madrid, Spain

**Keywords:** multiple sclerosis, biomarkers, neurofilaments, glial fibrillary acidic protein, physical exercise, Bayesian network meta-analysis

## Abstract

**Background:** The prognosis of people with multiple sclerosis (MS) has improved substantially in recent decades due to advances in diagnosis and treatment. Due to the unpredictable course and heterogenous treatment response in MS, there is a clear need for biomarkers that reflect disease activity in the clinical follow-up of these patients. We conducted a systematic review with Bayesian network meta-analysis with the aim of analyzing the effects of physical exercise on neurofilaments (NfL) and glial fibrillary acidic protein (GFAP) levels in patients with MS. **Methods:** A systematic review was conducted following the Preferred Reporting Items for Systematic Reviews and Meta-Analyses (PRISMA) guidelines, starting with a PICO (patient/population, intervention, comparison, and outcome) question: what are the clinical effects of physical exercise (with independence of the type) on NfL and/or GFAP levels in patients with MS compared with other interventions or no intervention whatsoever? A systematically comprehensive literature search was conducted from January to March 2024 to identify original studies that answered the PICO question, using the main data sources. The quality of the studies included was assessed using the Quality Index of Downs & Black. For studies included in the systematic review that followed a randomized controlled trial (RCT) design, the methodological quality of each paper was assessed using the Physiotherapy Evidence Database (PEDro) Scale. Risk of bias was also explored by two independent reviewers. Finally, all articles were classified according to the levels of evidence and grades of recommendation for diagnosis studies established by the Oxford Center for Evidence-Based Medicine. For continuous outcome measures with enough comparisons and a methodological quality greater than or equal to good according to the PEDro scale, a Bayesian network meta-analysis (NMA) was applied. The statistical analyses were performed in R (version 4.1.3, R Core Team 2023) using the “BUGSnet” and “gemtc” packages. Bayesian NMA can be used to obtain a posterior probability distribution of all the relative treatment effects, which allows us to quantify the uncertainty of parameter estimates and to rank all the treatments in the network. **Results:** Eight studies were included in this systematic review and six articles in the NMA, and they were appraised for quality. The characteristics of the included studies, types of training and described protocols, methodological quality, risk of bias, and clinical effects on the studied biomarkers were outlined. Qualitative synthesis, effects of different exercise modalities in NfL with the Bayesian NMA, selection of the final model and model assessment, and ranking of interventions are also shown. **Conclusions:** Our findings indicated that moderate-intensity exercise is more likely to reduce NfL concentration compared to high-intensity exercise, and, in turn, high-intensity exercise is more likely to reduce NfL concentration than low-intensity exercise. However, the effects of high-intensity exercise on GFAP levels were inconclusive.

## 1. Introduction

The prognosis of people with multiple sclerosis (MS) has improved substantially in recent decades due to advances in diagnosis and treatment [1]. There is an improvement in the survival of people with MS due to the early initiation of disease-modifying treatments. However, the overall mortality rate remains almost three-times higher than that of the general population, with an estimated loss of 13.1 years of life [2]. Factors influencing poor prognosis include demographic and environmental aspects, like advanced age, male sex, and low vitamin D levels, alongside clinical features such as primary progressive disease, brainstem or spinal cord involvement, and poor recovery from relapses. MRI findings, including T2 lesions, brain atrophy, and grey matter loss, further signal severe outcomes. Biomarkers such as oligoclonal bands, elevated neurofilament light chain, and retinal nerve thinning are also linked to worse progression [3]. Due to the unpredictable course and heterogenous treatment response in MS, there is a clear need for biomarkers that reflect disease activity in the clinical follow-up of these patients [4].

Neurofilaments (NfLs) are a group of neuronal intermediate filaments that play a crucial role in axonal growth and stability. Additionally, by integrating into various supramolecular structures, they contribute to synaptic organization and function within the central nervous system (CNS) [5]. Rosengren et al. [6], in the late 1980s, achieved the reliable quantification of the NfL in cerebrospinal fluid. NfL appears the most promising biomarker in MS patients, and there is now little doubt that Nfs should have a role in the follow-up of MS patients [5,6]. Elevated levels of Nfs in cerebrospinal fluid are associated with a higher rate of flares and lesions observed by magnetic resonance imaging, as well as being associated with disability and with the conversion from the relapsing–remitting to the secondary progressive form of MS [3].

Alternatively, glial fibrillary acidic protein (GFAP), an intermediate filament found in astrocytes analogous to NfL in neurons, has been suggested as a biomarker for assessing current disease progression and predicting future outcomes in MS [7,8]. Initial research measuring GFAP concentrations in the cerebrospinal fluid of MS patients revealed an association with neurological disability in the following years. Furthermore, high cerebrospinal fluid GFAP levels were associated with faster progression to an Expanded Disability Status Scale (EDSS) score of 3 and 6, and levels were higher in primary progressive MS than in relapsing–remitting MS [8]. Moreover, there is also evidence of increased GFAP levels in the cerebrospinal fluid of patients with progressive MS who had no recent relapses, showing the potential of GFAP levels for measuring pure progression. In contrast, although NfL was a sensitive indicator of neuroaxonal injury during acute disease activity (i.e., lesion formation and relapses), cerebrospinal fluid levels of GFAP remained unaffected in this state [9].

Other authors have shown that, using objective measurement methods, it is possible to stratify MS patients with good and poor prognosis over time (follow-up periods between 1 and 4 years), even though patients were stable in the indicated measurement period according to the commonly used clinical scales (EDSS) [10,11]. This unpredictable course in MS generates uncertainty in patients and their families, being the consultation about prognosis a recurrent issue that people with MS address with their physician [12,13].

For a long time, leading organizations discouraged physical exercise for individuals with MS, based on the assumption that it could aggravate fatigue or trigger disease exacerbations. However, evidence accumulated over the past years has demonstrated that carefully designed exercise programs are not only safe and feasible but also serve as an effective adjunctive therapy to mitigate symptoms [14]. Consequently, physical activity has emerged as a cornerstone of MS rehabilitation. Furthermore, exercise has been shown to exert beneficial effects on disease progression and modulate immune function [15]. Its pivotal role in managing MS has been extensively documented, particularly within rehabilitation strategies [16,17,18,19].

Exercise represents an effective rehabilitation strategy for individuals with multiple sclerosis, aiding in symptom management, functional recovery, quality-of-life enhancement, overall well-being, and increased engagement in daily activities [20]. While its role in improving functionality in people with MS is well established, evidence remains inconclusive regarding its impact on neurodegeneration, inflammation, and neuronal–axonal loss. Moreover, it does not prevent demyelination, and it does not alter the neurological course of the disease [21]. Further, its effects on biomarkers such as NfL and GFAP remain unclear. Therefore, we conducted a systematic review with Bayesian network meta-analysis with the aim of analyzing the effects of physical exercise on NfL and GFAP levels in patients with MS.

## 2. Methods

### 2.1. Design

This systematic review was conducted following the Preferred Reporting Items for Systematic Reviews and Meta-Analyses (PRISMA) guidelines [22], beginning with the formulation of a PICO question (patient/population, intervention, comparison, and outcome): what are the clinical effects of physical exercise (with independence of the type) on NfL and/or GFAP levels in patients with MS compared with other interventions or no intervention whatsoever?

This systematic review was registered in PROSPERO prior to its execution under reference number CRD42024608107.

### 2.2. Search Strategy

A comprehensive systematic literature search was performed between January and March 2024 to identify original studies addressing the PICO question. The search encompassed multiple databases, including PubMed, Scopus, Web of Science (WOS), ScienceDirect, the Physiotherapy Evidence Database (PEDro), and the Cochrane Library. Following the identification of eligible articles, a cross-referencing process was conducted to locate additional relevant studies. The specific search filters applied to each database are detailed in Table 1.

The combinations of keywords were ((Neurofilaments) AND (exercise)) AND (multiple sclerosis); and ((Glial Fibrillary Acidic Protein) AND (exercise)) AND (multiple sclerosis). The detailed search strategy for each database is shown in Table 2.

The search and screening of titles and abstracts were carried out independently by two authors to identify studies that satisfied the inclusion criteria. Any duplicates were excluded, and any discrepancies in study selection were addressed by consulting a third author for resolution.

### 2.3. Study Selection

This review considered studies published in English, with no restrictions on publication year, irrespective of their methodological design.

Exclusion criteria included studies unrelated to MS, study protocols, research involving animal models, poster presentations, conference or symposium abstracts, technical analyses with no clinical relevance, and studies that focused solely on healthy participants.

### 2.4. Participants

Only studies involving individuals with MS, considering their EDSS, regardless of MS type, were included in this review. The inclusion criteria were as follows: (a) individuals diagnosed with MS; (b) participants of both genders; (c) those engaged in a physical exercise program; and d) studies published without restrictions on the year of publication.

### 2.5. Interventions

For the papers included in this systematic review, the intervention group had to follow an exercise program (independently of the type: aerobic training, resistance training using or mixed) to study its effects on NfL and GFAP levels in patients with MS.

### 2.6. Outcome Measures

NfL and GFAP levels in patients with MS were registered as the main outcome measures for this systematic review, independently of the method (i.e., serum or plasma).

### 2.7. Data Extraction and Analysis

The search results were imported into the bibliographic citation manager Zotero, and duplicates were removed. Subsequently, two independent reviewers filled out an Excel template, recording the title and abstract of each citation for analysis. Full-text versions of the selected articles were then retrieved and reviewed by the research team to assess their relevance to the objectives of the review.

Data extracted from the studies included: study design, EDSS score and MS type, physical exercise protocol, sample size, dosage, outcome measures, and results. Two authors independently collected this information, and any disagreements were resolved through consensus, with a third author facilitating the discussion when necessary.

Once all the data were obtained, the papers were grouped into three different categories according to the intensity level of exercise described: “mild-intensity”, “moderate-intensity”, and “vigorous-intensity”. Exercise intensity was estimated according to the Metabolic Task Equivalent (MET), which has been used in healthy subjects and people with neurological disorders [23,24,25]:

Activities with low intensity are considered to fall within a range of 1.6 to 2.9 METs.

Activities of moderate intensity are categorized as those between 3.0 and 5.9 METs.

Activities requiring high intensity are those with a MET value of 6 or greater.

In those cases, in which the METS were not explicitly stated, METs values were estimated using the American College of Sports Medicine (ACSM) conversion tables and Herrmann et al. recommendations [23].

### 2.8. Assessment of Methodological Quality of the Studies and Risk of Bias

Historically, systematic reviews have primarily focused on randomized clinical trials (RCTs), often excluding observational studies due to the challenges in assessing their methodological quality. However, in many areas of healthcare, there is a lack of high-quality RCTs, and to ensure a critical and objective evaluation of studies outside this category, validated tools such as the Downs & Black Quality Index [26] are available. While the designs of RCTs, cohort studies, or case–control studies differ fundamentally, all require analysis of the intervention characteristics, confounding factors, and outcomes. In this systematic review, considering the types of studies included, the Downs & Black Quality Index was used to assess their methodological quality. The tool comprises 27 items, evaluating content quality (10 items), external validity (3 items), internal validity and bias (7 items), and confounding factors (6 items), along with statistical power (1 item). Higher scores reflect better methodological quality, with a maximum possible score of 27 points.

For studies included in the systematic review that followed a randomized controlled trial (RCT) design, the methodological quality of each paper was assessed using the Physiotherapy Evidence Database (PEDro) Scale, developed by The Centre of Evidence-Based Physiotherapy (CEBP). The PEDro Scale is a validated, reliable, and versatile 10-item tool designed to evaluate the quality of RCTs. It has been widely used as a measurement of methodological quality in systematic and literature reviews [27].

Two independent reviewers also utilized the Cochrane Handbook for Systematic Reviews of Interventions [28] to evaluate the risk of bias across six distinct domains: selection bias; performance bias; detection bias; attrition bias; reporting bias; other biases. Each study was independently evaluated, and it was categorized as having a “low risk of bias” if all domains were adequately addressed. If any domain was not properly addressed, the study was deemed to have a “high risk of bias”. Studies lacking sufficient information were considered to have an “uncertain” risk of bias. Discrepancies were resolved through discussion with a third reviewer. The RoB 2 tool was utilized to generate the necessary risk-of-bias diagrams [29].

In the final step, all included articles were classified according to the levels of evidence and the grades of recommendation for diagnostic studies, as defined by the Oxford Center for Evidence-Based Medicine [30].

### 2.9. Quantitative Synthesis: Bayesian Network Meta-Analysis

For continuous outcome measures with a sufficient number of comparisons and a methodological quality rated as “good” or higher based on the PEDro scale, a Bayesian network meta-analysis (NMA) was conducted. Statistical analyses were performed using R software (version 4.1.3, R Core Team 2023) with the “BUGSnet” and “gemtc” packages. The Bayesian NMA approach provides posterior probability distributions for the relative treatment effects, enabling the quantification of uncertainty in parameter estimates and the ranking of all treatments within the network. The analysis was conducted among exercise interventions in people with multiple sclerosis (MS). Each node represents a type of rehabilitation treatment categorized by exercise intensity, high-intensity training, low-intensity training, and moderate-intensity training, according to the Metabolic Task Equivalent (MET). Conventional physiotherapy or rehabilitation treatments were classified as low-intensity training, which acted as the reference group. The NMA was performed only for biomarkers with a sufficient number of comparable studies. Other characteristics that could influence the effectiveness of the therapy were not included in the analysis; instead, individual studies were described separately, detailing patient characteristics and types of interventions. Treatments with similar intensities were represented within the same treatment arm.

The connectivity between nodes was determined based on the direct comparisons available in the literature. If two treatments had been directly compared in the studies, they were linked in the network. Treatments that had no direct comparisons were not connected. This connectivity structure was validated using the node-split method to assess the consistency between direct and indirect comparisons. If discrepancies were detected in the plot comparing consistency and inconsistency models, an additional meta-regression model was applied, including age and baseline scores as covariates. This approach ensured that only reliable evidence supported the connections between nodes and helped detect any inconsistencies in the model.

The Bayesian NMA was performed using the Markov Chain Monte Carlo (MCMC) simulation algorithm with Gibbs sampling. For each outcome measure, the mean difference (MD) was calculated as the effect size. When results were reported as 95% confidence intervals, data were converted into standard deviations (SD) using appropriate formulas. Treatment effectiveness was assessed using the surface under the cumulative ranking curve (SUCRA) and by inspecting the rankogram, where higher values indicate a greater probability of effectiveness for each treatment. Direct comparisons between treatments were analyzed as MD with 95% credible intervals (CIs) presented in a league table and forest plots, when statistically significant differences were identified.

In each NMA, we evaluated the choice of a random or fixed effects model and assessed the presence of inconsistency. This was achieved by analyzing the leverage graph (defined as the posterior mean residual deviance of each study arm minus the deviance at the posterior mean of the fitted values) in relation to the Bayesian residual deviance (w), which reflects each study arm’s contribution to the model’s posterior mean deviance. Additionally, we examined the deviance using the mean residual deviance (Dres), the effective number of parameters or sum of leverages (pD), and the Deviance Information Criterion (DIC), which is the sum of Dres and pD. In this context, lower values indicate a better model fit.

When a variable had sufficient comparisons, the presence of inconsistency was evaluated using the node-split method, which helps identify significant differences between direct and indirect comparisons. Furthermore, if discrepancies were detected in the plot of posterior mean values comparing consistency and inconsistency models, an additional meta-regression analysis was conducted, incorporating age and baseline scores as covariates. To assess the convergence of posterior distributions for the parameters, Markov Chain Monte Carlo (MCMC) sampling was used, with trace plots of the mean for each comparison across iterations, along with density plots for the effect size estimates being examined.

Publication bias was evaluated using the D measure based on the Robust Bayesian Copas selection model, with thresholds defined as negligible (<0.25), moderate (0.25–0.5), high (0.5–0.75), and very high (>0.75).

## 3. Results

**Figure 1:**
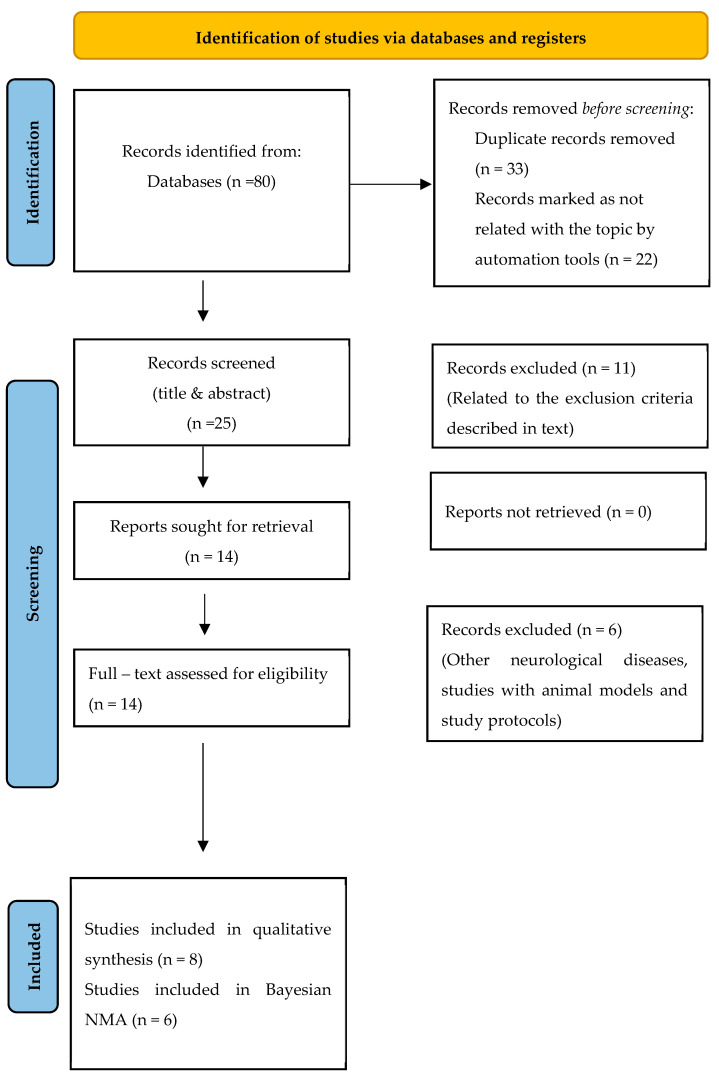
Flow chart of the identified studies according to the PRISMA 2020 Statement.

The database searches initially identified 80 papers, of which 55 were discarded prior to screening, primarily due to duplicates. Following the initial screening of titles and abstracts, 11 records were excluded for not meeting the inclusion criteria. Ultimately, 14 records were evaluated for eligibility, and 6 were excluded [31,32,33,34,35,36] for various reasons, including study protocols, focus on other neurological disorders, and the use of animal models. Finally, eight studies [37,38,39,40,41,42,43,44] were included in the systematic review and six articles in the NMA [37,38,39,41,43,44], and these were appraised for quality (Figure 1). The studies conducted by Mulero et al. [40] and Amiri et al. [42] were excluded from the NMA due to receiving a score below “good” on the PEDro scale. The network meta-analysis was performed only on studies assessing NfL concentrations, and studies assessing GFAP concentrations were limited.

### 3.1. Characteristics of Included Studies

A total of eight studies with 338 patients (range from 11 to 86 patients) were included in this systematic review. The studies showed a greater predominance of women than men. Further, 165 individuals presented a relapsing–remitting MS (RRMS), with the rest with progressive forms of the illness. The mean disease duration was 13.07 years, and the mean in the EDSS was 2.43 (with a range from 0 (normal neurological exam, no disability in any functional system) to 4.59 (significant disability but up and about much of the day, able to work a full day, may otherwise have some limitation of full activity or require minimal assistance, able to walk without aid or rest for 300 m) scores). All papers focused on aerobic training, but one studied the relationship between the biomarkers studied and functional mobility in people with MS [42]. All studies came from Europe and Asia.

NfL was studied in all papers included [37,38,39,40,41,42,43,44], while GFAP was studied in [37,38]. The clinical characteristics of the included studies are shown in Table 3.

### 3.2. Type of Training and Protocols Described

All included papers calculated and described the exercise intensity of the experimental group. Gravesteijn et al. [37], Ercan et al. [38], Joisten et al. [39], and Langeskov et al. [44] employed aerobic exercise for the experimental group. Mulero et al. [40], Balagi et al. [41], Amiri et al. [42], and Maroto et al. [43] applied a muscle strengthening protocol at different %1RM ranges.

Control groups received a nurse control intervention [37,43], home exercises [38], or moderate continuous training [39] to maintain their usual rhythm of life [41,42]. Langeskov-Christensen et al. [44] employed, as a control intervention, physiotherapy treatment within that usual rhythm. Mulero et al. [40] presented a case-series study so no control groups were recruited to compare their findings (Table 4). The detailed physical exercise protocols applied in the included studies are shown in Table 3.

The interventions were grouped as follows:

- Light intensity [37,38,39,41,42,43,44]:

The activities proposed were mostly related to not practicing any sports activity but maintaining a normal lifestyle [41,42,43], a nursing control (37), lumbopelvic mobility exercises (38), or conventional physiotherapy sessions [44].

All protocols had an intensity equal to or greater than 1.5 METs and less than 3 METs.

- Moderate intensity [39,41,42]:

Jonstein et al. [39] developed an aerobic exercise intervention 3 times a week for 3 weeks of 24 min per session with an intensity of 65% of maximum heart rate, as well as a 3 min warm-up and cool-down period. Balagi et al. [41] applied two 60 min intervention groups through the Pilates method with 10 min warm-up followed by a 40 to 45 min workout and a cool-down period with the remaining time to 60 min. Amiri et al. [42] performed a strengthening exercise protocol for 45 to 60 min 3 times per week for 8 weeks. All sessions began and ended with a 10 min general warm-up and cool-down, and a 3 min specific warm-up was also performed. The protocol included 10–12 repetitions at an intensity of 45% of the 1 repetition maximum (1RM) during the first eight interventions, 10–12 repetitions at an intensity of 50% 1RM during the following eight interventions, and 10–12 repetitions at an intensity of 55% 1RM.

All interventions in this category had an intensity from equal to or greater than 2 METs and less than 6 METs.

- High intensity [37,38,39,40,43,44]:

Gravejstein et al. [37] applied an aerobic exercise protocol with a cycloergometer, 3 times a week for 16 weeks, for 4 weeks supervised by a professional and for 12 weeks at home. Six 3 min intervals were performed at an intensity of 40% of maximum power, 1 min at 60% of maximum power, and 1 min at 80% of maximum power. Ercan et al. [38] performed an aerobic home-exercise protocol in combination with lumbar stabilization exercises 3 times a week for 8 weeks. The aerobic exercise consisted of a 5 min warm-up at an intensity of 20% of VO_2_ max, 30 min of exercise at an intensity of 60–70% of VO_2_ max. Johnstein et al. [39] presented an intervention group performing three sessions per week for 3 weeks, composed of five high-intensity intervals of 1.5 min at an intensity of 95–100% of maximum heart rate with a 2 min rest between intervals. Langeskov-Christensen et al. [44] developed a protocol of two sessions per week for 24 weeks in a supervised manner. Interventions started with a duration of 30 min and an intensity of 65% of maximum heart rate, progressing up to 60 min duration and an intensity of 95% of maximum heart rate. Mulero et al. [40] and Maroto-Izquierdo et al. [43] applied a protocol of three weekly sessions during 6 weeks of strengthening exercise. The interventions consisted of eight exercises of three sets of 8 to 10 repetitions with a rest of 2 between sets but not between exercises. Intensity started at 70% of 1RM and progressed to 80% of 1RM. Additionally, Mulero et al. [40] included a 5 min aerobic exercise on an elliptical bike as a warm-up. The exercise was always supervised by one or more experts.

All interventions in this category presented an intensity equivalence equal to or greater than 6 METs.

### 3.3. Methodological Quality of the Studies and Risk of Bias

The methodological quality of the studies is shown in Table 5. The studies ranged from 12 to 21 points in the Quality Index of the Downs & Black tool. Most of the studies presented problems in the items related to internal validity, particularly with the randomization, blinding, and confounding factors. A higher score was reached by Gravejstein et al. [37] and Langeskov-Christensen et al. [44], and the lowest score was reached by Mulero et al. [40] and Amiri et al. [42]. The PEDro scale was administered with a range from 0 points or not applicable up to 9 points. The lowest score was obtained in the research of Mulero et al. [40], not being possible to award any points due to the absence of a control group in their research, and the highest score was obtained by Jonstein et al. [39], with a total of 9 points.

**Figure 2:**
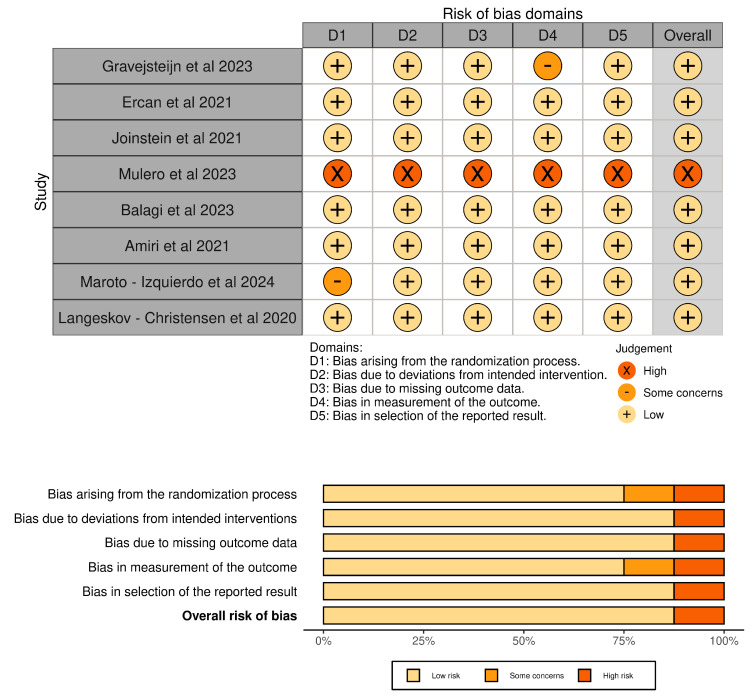
Traffic light plot and summary plot.

The risk of bias of the papers included in the systematic review is shown in Figure 2.

Levels of evidence and grades of recommendation of the papers included are shown in Table 5. Gravesteijn et al. [37], Ercan et al. [38], Joisten et al. [39], Balagi et al. [41], Amiri et al. [42], Maroto-Izquierdo et al. [43], and Langeskov–Christensen et al. [44] reached a 2b score (low-quality randomized controlled trial due to a limited sample size and no follow-up assessments). Mulero et al. [40] reached a 4c score (low-quality cohort and case–control studies).

### 3.4. Clinical Effects on the Biomarkers Studied: Qualitative Synthesis

Gravesteijn et al. [37] recruited 55 MS patients randomized to an experimental group that received aerobic training and to a control group that received a nurse control intervention. No between-group differences were found for any biomarker (NfL and GFAP). The authors concluded that the aerobic exercise protocol employed did not result in statistically significant changes in the tested neuro-specific blood-based biomarkers in people with MS.

Ercan et al. [38] recruited 38 MS patients randomized to an experimental group that received aerobic exercises and home exercises versus a control group that only practiced the same home exercises. NFL and GFAP levels were statistically lower in the study group at the end of the study than before the study. In the control group, no significant changes were observed in serum NFL and GFAP levels.

Joisten et al. [39] recruited 38 MS patients randomized to an experimental group that received high-intensity interval training (HIIT) compared to a control group that practiced moderate continuous training (MCT). Their findings showed that acute exercise reduced NfL in plasma and HIIT consistently led to greater effects than MCT in people with MS.

Mulero et al. [40] included 11 participants with MS in their study. Following a resistance training program, the median plasma NfL levels significantly dropped from baseline (6.61 pg/mL) to 1 week after the intervention (4.44 pg/mL). This reduction was maintained even after 4 weeks of detraining (4.38 pg/mL). These findings may indicate a potential neuroprotective effect of resistance training in individuals with MS.

Balagi et al. [41] included 44 women diagnosed with RRMS in their study. They designed a physical exercise protocol based on the Pilates method, with each session consisting of a 10 min warm-up followed by 40 to 45 min of various movements. The results revealed significant differences between the groups in serum levels of NfL, vitamin D, and Fatigue Severity Scale (FSS) scores after completing 8 weeks of both OPT and HPT training across all groups (*p* < 0.001).

Amiri et al. [42] recruited 24 women with MS, and they performed a muscle strengthening program of 45–60 min three times per week for 8 weeks. According to the ANCOVA test, the 8-week resistance training significantly reduced the serum Tau protein level (*p* < 0.0001) but had no significant effect on NFL levels (*p* = 0.110) compared to the control group.

Maroto-Izquierdo et al. [43] recruited 11 people with MS who received a high-intensity strengthening exercise protocol. Statistically significant differences were found between the experimental and control group (39.6, *p* = 0.005, t (10) = 3.5, ES = 1.07).

Langeskov-Christensen et al. [44] obtained a sample of 64 people with MS randomized to an intervention group that performed high-intensity aerobic exercise. They found no significant differences between the two groups in plasma NfL.

### 3.5. Effects of Different Exercise Modalities in NfL: Bayesian NMA

**Figure 3:**
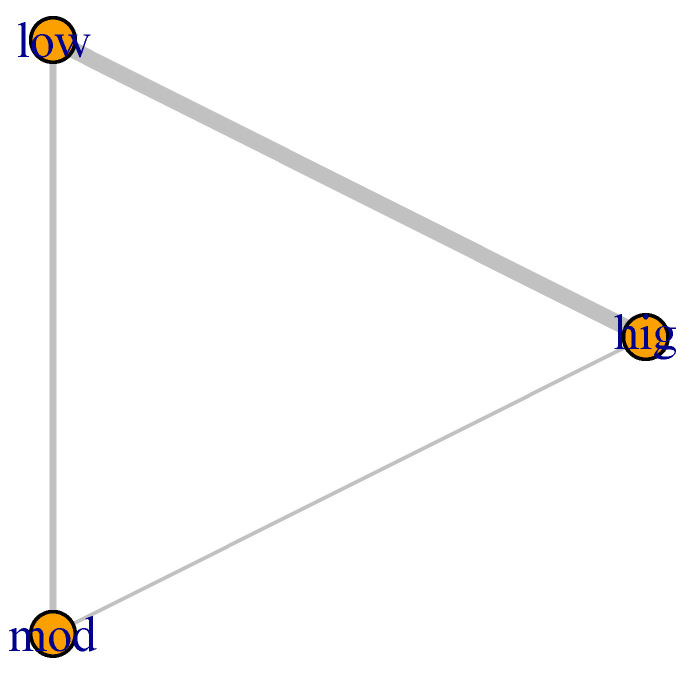
Network diagram of the effect of interventions on NfL levels. The width of each line is proportional to the number of trials comparing every pair of treatments, and the size of each circle is proportional to the number of randomly allocated participants (sample size). Hig: High-Intensity Training; Low: Low-Intensity Training; Mod: Moderate-Intensity Training.

Six studies [37,38,39,41,43,44] were included in the Bayesian NMA with effects of three possible comparisons (three types of interventions) on NfL levels in pwMS after exercise (n = 281). The exercise modalities were categorized as high-intensity exercise [37,38,39,40,43,44], moderate-intensity exercise [39,41,42], and low-intensity exercise (interventions) [37,38,39,41,42,43,44]. The comparisons were diverse, with some authors comparing high-intensity exercise vs. low-intensity exercise (four comparisons); high-intensity exercise vs. moderate-intensity exercise (one comparison); and moderate-intensity exercise vs. low-intensity exercise (two comparisons). The network formed by the different comparisons can be seen in Figure 3. In the analysis conducted, low-intensity exercise was taken as the reference group.

### 3.6. Selection of the Final Model and Model Assessment

The random effects model was the best fitting model for the analysis, which did not present leverage outliers. Bayesian models were used to analyze the effects of exercise on NfL levels. To ensure model robustness, simulations with Markov Chain Monte Carlo (MCMC) were performed, using different numbers of iterations to verify algorithm convergence. Initial simulation (MCMC1): A few iterations were conducted to observe the initial trend of the estimate. Extended simulation (MCMC2): The number of iterations was increased to improve estimation accuracy and verify convergence. A convergence evaluation was conducted through trace plots (time series), density plots to compare distribution normality, and calculation of the multivariate potential scale reduction factor (MPSRF) to verify global convergence.

In the first model, the chains showed a slight divergence in their trends, indicating a possible lack of convergence. In the second model, better convergence was observed, with random fluctuations and a PRSF close to 1 (1.000213), indicating a good model fit, which led to basing the study results and interpretations on this second model’s outputs (Supplementary Material).

**Figure 4:**
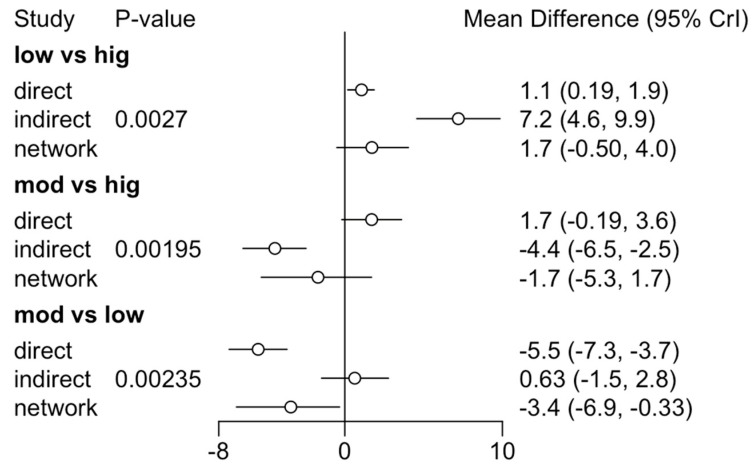
Direct and indirect comparison of interventions on NfL levels in network meta-analysis. Evaluates the consistency between direct and indirect comparisons for a specific pair of treatments. Circles: Point estimates of treatment effect for direct and indirect comparisons. Lines: 95% confidence intervals. When the lines cross 0, it indicates that there are no significant differences between the compared treatments. If the direct and indirect comparisons differ markedly, it may indicate inconsistency in the network.

In all three comparisons (high vs. low, high vs. moderate, and low vs. moderate), node-splitting analysis detected significant inconsistencies between direct and indirect evidence (−5.5 vs. 0.64), which could indicate that studies comparing treatments directly are not consistent with those allowing for indirect comparisons through other nodes in the network (Figure 4).

### 3.7. Classification of Interventions Based on Effectiveness on NfL Levels

**Figure 5:**
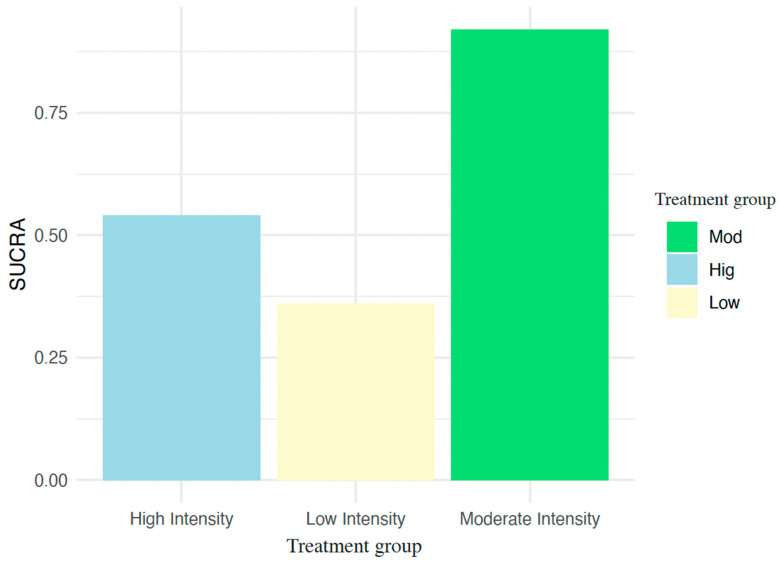
Surface under the cumulative ranking curve. Higher SUCRA (surface under the cumulative ranking curve) values (close to 100%) indicate the probability that a treatment is more successful in reducing NfL levels compared to other treatments evaluated.

The interventions with the greatest effect on NfL levels in people with MS (Figure 5) were moderate-intensity training (SUCRA = 92.24, credibility interval 75.29–95.19), followed by high-intensity training (SUCRA = 54.04, credibility interval 50.08–89.05) and low-intensity training (SUCRA = 3.6, credibility interval 29.81–89.01). According to the rankogram (Supplementary Material), the most likely best intervention for the MoCA was moderate-intensity training (86.21%), followed by high-intensity training (12.11%) and low-intensity training (0.63%).

The differences between high and low intensity were 1.73 (1.12), 95% credibility interval: [−0.5091, 4.017], indicating that, on average, high-intensity exercise resulted in an increase of 1.73 units in GFAP levels compared to low-intensity exercise. The differences between high and moderate intensity were −1.72 (1.73), 95% credibility interval: [−5.32, 1.7], indicating that moderate-intensity exercise resulted in an increase of 1.72 units in GFAP levels compared to high-intensity exercise. The differences between moderate and low intensity were 3.45 (2.06), 95% credibility interval: [2.31, 4.82], indicating that moderate-intensity exercise resulted in a reduction of 3.45 units in NfL levels compared to low-intensity exercise (Figure 4).

## 4. Discussion

We conducted a Bayesian network meta-analysis on the effect of different exercise modalities on NfL and a systematic review of different exercise modalities on NfL and GFAP levels. Our findings indicated that moderate-intensity exercise is more likely to reduce the NfL concentration compared to high-intensity exercise, and, in turn, high-intensity exercise is more likely to reduce the NfL concentration than low-intensity exercise; however, the effects of high-intensity exercise on GFAP levels were inconclusive.

The development and validation of biomarkers capable of objectively assessing the clinical status of MS patients remain a need and a challenge. Overall, an ideal biomarker for MS should be able to (a) facilitate diagnosis and/or establish the disease phenotype; (b) contribute to prognosis; (c) assess response to treatment [45].

The biomarkers studied in our systematic review are associated with inflammatory activity and secondary axonal damage, with disease severity and the occurrence of flares. Likewise, a significant relationship has been described between higher NfL levels and higher disease activity, as evidenced by MRI and a higher degree of brain atrophy [45]. This justifies the importance of knowing the effects of physical exercise on NfL and GFAP levels.

There are a multitude of biomarkers linked to MS that can be highlighted: biomarkers of axonal damage: NfL, Tau protein, Amyloid-Precursor Protein (APP), Tubulin β (TUB β); biomarkers of neural damage: 14-3-3 Protein, Neuron-Specific enloase (NSE); biomarkers of glial damage: GFAP, S100β Protein, Nitric Oxide (NO) [46]. Parallel to these, immunomodulatory or inflammatory markers should be highlighted: a) proinflammatory: Interleukin 17 (IL-17), Interferon α (IFN-α)- and Tumor Necrosis Factor (TNF)-α; b) anti-inflammatory: interleukins IL-10 and IL-4. Other biomarkers of relevance different from the previous categories include the following: neurotrophins, blood–brain barrier function markers (BBB) and alterations in tryptophan (Trp) metabolism, expressed by the Kyn/Trp ratio [47,48].

In the present investigation, we found conflicting evidence regarding the effect of exercise on GFAP levels. The studies by Gravesteijn et al. [37] and Ercan et al. [38] involved a HIIT intervention model in the former and continuous high-intensity aerobic training in the latter. The first mentioned investigation found no statistically significant differences in relation to GFAP levels, whereas the latter did find statistically significant changes in the concentration of this biomarker. The findings of the research by Gravejstein et al. [37] are not in line with the systematic review with meta-analysis by Youseff et al. [49], who observed how both a moderate-intensity aerobic exercise model and a high-intensity HIIT model showed statistically significant changes. In turn, the study by Joisten et al. [50] found that HIIT aerobic exercise had a greater effect than a moderate-intensity protocol on the decrease in different biomarkers: neutrophil-to-lymphocyte ratio (NLR); systemic immune-inflammation index (SII). However, the research by Ercan et al. [38] presented findings similar to those presented above. The difference we found between the research results of Gravejstein et al. [37] and Ercan et al. [38] could be due to an unsupervised home-based HIIT-type intervention, which may be more difficult to execute than a continuous high-intensity aerobic exercise protocol.

Regarding the effect of physical exercise on NfL levels, moderate-intensity physical exercise was more likely to decrease the NfL concentration than high-intensity and low-intensity exercise. With respect to the greater therapeutic potential of high-intensity versus low-intensity exercise, these results would be expected due to the neuroprotective and anti-inflammatory effect of physical exercise [51], and it is possible to expect that the greater the amount or intensity of an exercise, the greater its effect. However, a comparison of high-intensity versus moderate-intensity exercise did not seem to show the same effects. The decrease in the effect of high-intensity versus moderate-intensity exercise could be justified by the chronoprogramming of the investigations of Gravejstein et al. [37] and Ercan et al. [38], in which presenting an intervention protocol without a timely follow-up could have led to an inadequate execution of the intensity, thus compromising the results, as we previously exposed with the levels of GFAP. On the other hand, the duration of two of the investigations in the moderate-intensity group [41,42] was longer than two of the supervised investigations in the high-intensity group [40,43]. In turn, most of the subjects who showed progressive forms of presentation were particularly concentrated in the high-intensity group, there being the possibility that the neurodegeneration of the disease itself attenuated the therapeutic potential of the different interventions, highlighting the investigations of Gravejstein et al. [38] and Langeskov-Christensen et al. [44], who showed 16 and 24 weeks of intervention, respectively.

There is other research about the effect of exercise on different biomarkers in people with MS, showing a positive effect on most of them. Negaresh et al. [47] found positive effects of physical exercise on BBB and different neurotrophins such as BDNF, NGF, and Neurotrophin–4. In turn, it generated a decrease in different proinflammatory markers in people with MS. Wong et al. [52] found that 8 weeks of aerobic and strengthening exercise could reduce the concentration of IL-17 in people with RRMS, and an aerobic exercise protocol could decrease the concentration of IFN-α in people with PPMS and PPMSD.

On the other hand, the results of the Bayesian network meta-analysis seem to indicate that the three comparisons performed present significant inconsistencies in both direct and indirect comparisons. These findings may be due to the great heterogeneity of the different studies.

We found heterogeneity in terms of the samples recruited in the different studies. Although most of the subjects presented RRMS, subjects with progressive courses of the disease were mostly concentrated in the studies that were part of the high-intensity group, so the effect of this training modality may have been underestimated in people with RRMS and the effect of moderate-intensity exercise overestimated in people with progressive courses of the disease. On the other hand, two [41,42] of the three studies that were part of the moderate-intensity group presented a longer protocol duration than different investigations of the high-intensity group, such as the investigations of Maroto-Izquierdo et al. [43], which may have presented better results due to their longer intervention time.

As previously mentioned, different investigations of the high-intensity group [37,38] and one article of the moderate-intensity group [41] did not present supervision during the entire protocol, so the dose prescribed in these investigations could have been altered. In turn, it should be noted that although all the intervention groups were established based on an objective categorization (METs), it is to be expected that aerobic physical exercise [37,38,39,44] may generate a different effect to that produced by strengthening exercise, even though they are in the same range in this categorization [40,41,42,43].

On the other hand, two investigations [41,42] measured the concentration of NfL through the ELISA system instead of the SIMOA system, used in the other investigations, this being the gold standard for the identification of NfL by showing greater sensitivity than the former [53,54].

On the other hand, some of the studies included in the NMA presented low methodological quality according to the D&B scale [42,43], highlighting the absence of control of different confounding factors that could negatively affect the results of the interventions. The levels of evidence and grades of recommendation of the papers included ranged from 2b to 4c. Finally, it should be noted that these biomarkers studied in the present systematic review were not specific to MS, as they are also released in other conditions with active neuroaxonal loss, such as head injuries or exclusively neurodegenerative diseases [55].

This study has several limitations. Despite conducting a comprehensive literature search and employing rigorous methods for study selection and data extraction, publication bias cannot be ruled out, as only articles published in journals were included based on our criteria. Additionally, given the variability in the interventions and dosages used, our findings should not be generalized to all MS patients, other neurological conditions, or different biomarkers. An ideal protocol of exercise in MS patients cannot be offered based on this systematic review, due to the different modalities explored (aerobic or resistance training, but not a combination of them), particularly with regard to low-intensity physical activity, since the studies that formed part of this group showed less homogeneity in the activities carried out compared to the other two groups (moderate and high intensity). Further, our findings cannot be extrapolated to other EDSS scores (different from 0 to 4.5 points) and always under professional supervision. Finally, the heterogeneity of the methodological quality of the studies, the small samples, and the high risk of bias are aspects that lead to a cautious interpretation of the results.

## 5. Conclusions

Well-structured exercise programs seem to be feasible, safe, and useful and present an impact on the biomarkers studied in this study (NfL and GFAP). Our findings indicated that moderate-intensity exercise is more likely to reduce the NfL concentration compared to high-intensity exercise, and, in turn, high-intensity exercise is more likely to reduce the NfL concentration than low-intensity exercise. However, the effects of high-intensity exercise on GFAP levels were inconclusive.

## Figures and Tables

**Table 1:** Search filters in database.

Database	Search Filter
Pubmed	Availability: full text Publication date: without restriction of year Document type: Clinical trial or Randomized Controlled Trial
Scopus	Publication date: without restriction of year Language: English Document type: Clinical trial or Randomized Controlled Trial
Web of Science	Publication date: without restriction of year Language: English Document type: Clinical trial or Randomized Controlled Trial
ScienceDirect	Publication date: without restriction of year Language: English Document type: Clinical trial or Randomized Controlled Trial Subject area: neuroscience
Physiotherapy Evidence Database	No filter
Cochrane Library	Publication date: without restriction of year

**Table 2:** Search keywords and equation.

Database	Search Equation
PubMed	((“Neurofilaments”[Title/Abstract] OR “Glial Fibrillary Acidic Protein”[Title/Abstract]) AND (exercise[Title/Abstract])) AND (“multiple sclerosis”[Title/Abstract])
Scopus	((“Neurofilaments”[Title/Abstract] OR “Glial Fibrillary Acidic Protein”[Title/Abstract]) AND (exercise[Title/Abstract])) AND (“multiple sclerosis”[Title/Abstract])
Web of Science	TS = ((“Neurofilaments” OR “Glial Fibrillary Acidic Protein” AND (exercise)) AND (“multiple sclerosis”))
ScienceDirect	((“Neurofilaments” OR “Glial Fibrillary Acidic Protein” AND (exercise)) AND (“multiple sclerosis”))
Cochrane Library	((“Neurofilaments” OR “Glial Fibrillary Acidic Protein” AND (exercise)) AND (“multiple sclerosis”))

**Table 3:** Clinical characteristics of the included studies.

Study	Study Desing	Location	Sample	MS Subtype	Disease Duration in Years (Median (IQR))	EDSS (Median (IQR)	Male/Female	Age, Mean ± SD	Biomarkers Studied
Gravesteijn et al. [37]	Secondary analysis of a randomized controlled trial	The Netherlands	55 MS patients: n = 30 (aerobic training) n = 25 (control group)	RRMS, 17 (57) PMS, 8 (27) Unknown, 5 (17) RRMS, 16 (64) PMS, 8 (32) Unknown, 1 (4)	7 (4; 9) 12 (3; 19)	2.5 (2.0–3.0) 3 (2.0–3.5)	8/22 8/17	43.5 (10.1) 48.1 (10.6)	NfL, GFAP
Ercan et al. [38]	Randomized controlled trial	Turkey	38 MS patients: n = 19 (aerobic exercises and home exercises) n = 19 (home exercises)	RRMS RRMS	8.26 (3.03) 7.26 (3.89)	1.90 (1.11) 2.05 (0.98)	-- --	27.84 (4.99) 29.58 (5.97)	NfL, GFAP
Joisten et al. [39]	Secondary analysis of a randomized controlled trial	Germany	69 MS patients: n = 35 (HIIT) n = 34 (MCT)	RRMS, 21 PMS, 14 RRMS, 21 PMS, 13	14.0 (8.26) 12.24 (7.5)	4.44 (1.06) 4.59 (1.08)	11/24 15/19	50.89 (10.31) 49.65 (10.04)	NfL
Mulero et al. [40]	Longitudinal and interventional study	Spain	11 MS patients	RRMS, 11	10.6 (6.8)	0 (range: 0–2)	2/9	40.8 (7.8)	NfL
Balaghi et al. [41]	Randomized trial study with a pretest-posttest	Iran	44 MS patients: Home-based training Outdoor training Control group	RRMS, 44	--	-- (inclusion criteria between EDSS 2–5)	44 female	35.75 (5.15) 35.16 ± 4.44 35.5 (3.62)	NfL
Amiri et al. [42]	Quasi–experimental	Iran	24 MS patients: Resistance training (n = 12) Control group (n = 12)	--	--	-- (inclusion criteria between EDSS 2–5)	24 female	-- --	NfL
Maroto- Izquierdo et al. [43]	Longitudinal counterbalanced pilot study (a series of cases)	Spain	11 MS patients	11 RR	12. (6.7)	0.5 (0.8)	81.8% female	40.8 (7.8)	NfL
Langeskov-Christensen et al. [44]	Randomized controlled trial	Denmark	86 MS patients: PAE (n = 43) Control group (n = 43)	PAE group: 95% RR 5% PP 0% SP Control Group: 79% RR 9% PP 12% SP	PAE group: 10.9 (7.9) Control group: 8.6 (6.0)	PAE group: 2.7 (1.4) Control group: 2.8 (1.6)	PAE group: 60% female Control group: 60% female	PAE group: 44.0 (9.5) Control group: 45.6 (9.3)	NfL

**Table 4:** Physical exercise protocols in the included studies.

	Experimental Group	Control Group
Gravesteijn et al. [37]	Training sessions on a cycle ergometer three times a week, consisting of 6 intervals of 3 min at 40% of PP, 1 min at 60% of PP, and 1 min at 80% of PP, during a period of 16 weeks. In total, 12 sessions were conducted in an outpatient clinic under supervision of an experienced physiotherapist, whereas the remaining 36 sessions were home-based using identical equipment as provided by the study team for the duration of the intervention.	Nurse control intervention had three 45 min sessions with an experienced MS nurse over the course of the 16-week intervention period. During these sessions the MS nurse informed participants about MS-related fatigue and patient concerns were discussed. During the intervention period patients were not referred to any other facility for the treatment of their fatigue.
Ercan et al. [38]	The patients in the study group were given home exercises 3 times a week and aerobic exercise with an electronic cycle ergometer for 8 weeks. Each exercise bout was applied as: 5 min of warm-up = at 20% of VO_2_ max; 30 min of exercise = 60–70% of VO_2_ max; 5 min cooling period. The home exercise protocol was the same as the control group.	Patients were prescribed home exercises three times a week for a duration of 8 weeks. The program included four distinct lumbar stabilization exercises, tailored to the individual’s functional and motor abilities, with each exercise performed for 4–5 repetitions. A 2 min rest was recommended between sets. On average, each session lasted between 15 and 20 min. The exercises were designed according to the patient’s functional status, incorporating combinations of alternating and slow or fast movements performed in lying, sitting, and standing positions. Instructions for the exercises were provided in clear detail. Patients were scheduled for biweekly clinic visits to monitor their exercise execution and assess progress within the protocol.
Joisten et al. [39]	The intervention consisted of a 3-week training program during inpatient rehabilitation, with 3 exercise sessions per week. Exercise intensity for each participant was determined based on the maximum heart rate reached during an incremental exercise test to exhaustion, conducted prior to the intervention. Each session in both groups included a 3 min warm-up and cool-down period. In the HIIT group, participants performed 5 high-intensity intervals lasting 1.5 min at 95–100% of their maximum heart rate, followed by 2 min of unloaded pedaling between intervals.	3-week training intervention during inpatient rehabilitation with 3 exercise sessions per week MCT group exercised continuously for 24 min at an intensity of 65% of the maximum heart rate.
Mulero et al. [40]	The training program lasted 6 weeks, consisting of 18 circuit-based resistance sessions with a 48 h rest period between sessions. Each session included 3 sets of 8–10 repetitions, incorporating both concentric and eccentric muscle actions across 7 exercises targeting all major muscle groups. Exercise intensity was gradually increased every 2 weeks, ranging from 70% to 80% of the one-repetition maximum. A 2 min rest interval was provided between sets. The individual exercise intensity was determined using the ACSM protocol to estimate the one-repetition maximum, calculated 1 week prior to the intervention. Additionally, each session began with a 5 min warm-up on an elliptical bike at a moderate perceived intensity.	--
Balaghi et al. [41]	The training groups engaged in 60 min of Pilates exercises three times a week for 8 weeks, either at home or outdoors, with at least 48 h of rest between sessions. Instructions were provided through a DVD. Each session began with a 10 min warm-up consisting of seven movements, followed by 40 to 45 min of main body exercises incorporating 14 movements, and concluded with a cool-down phase featuring 9 movements. The outdoor group performed only the main body training. Training intensity was monitored using the Borg rating of perceived exertion scale and heart rate. In the first two weeks, participants completed four repetitions of each Pilates movement. Repetitions were gradually increased every two weeks, reaching 10 repetitions by the final two weeks. Adherence, fidelity, and compliance were tracked through self-reported exercise diaries, completed immediately after each session. In addition, a weekly phone call was conducted to confirm details of frequency, intensity, duration, any challenges encountered during exercise, and any adverse events.	Subjects in the control did not participate in any sports activity and received their conventional care supervision.
Amiri et al. [42]	The training group engaged in 45 to 60 min of resistance training, three times a week, over an 8-week period. Each session began with a 10 min general warm-up, consisting of slow walking, stretching, and flexibility exercises, followed by 3 to 5 min of specific warm-up activities, and concluded with 10 min of cool-down. The training protocol included exercises such as leg press, leg extension, leg curl, bench press, lat pull-down, lateral raise, triceps pushdown, arm curl, and two basic abdominal crunch variations (21). To regulate exercise intensity, participants performed two sets of 10–12 repetitions for each exercise, starting at 45% of their one-repetition maximum (1-RM) during the first eight sessions. The intensity was increased to 50% of 1-RM for the second set of eight sessions and 55% of 1-RM for the final eight sessions. A 1–2 min rest period was given between sets. Participants also rested for 2 min between different exercises. It is important to note that participants’ 1-RM values were reassessed every two weeks for all exercises.	Control subjects did not partake in any sports activity and received their conventional care supervision.
Maroto- Izquierdo et al. [43]	Subjects in the training group performed 18 high-intensity circuit-based resistance training sessions over 6 weeks. Each session-initiated with 5 min moderate-intensity warm-up on an elliptical machine and included three sets of 8–10 repetitions of seven exercises targeting all major muscle groups. The intensity of the exercise started at 70% 1-RM and progressed to 80% 1-RM. The rest between sets and training sessions was 2 min and 48 h respectively.	Control subjects did not partake in any sports activity and received their conventional care supervision avoiding any intense exercise, nevertheless, it was recommended 30 min sessions of moderate intensity aerobic exercise three times per week.
Langeskov-Christensen et al. [44]	The experimental group underwent 24 weeks of PAE twice weekly between 1:00 AM and 6:00 PM, comprising one continuous and one interval exercise session. Exercise volume increased from 30 to 60 min and intensity increased from 65 to 95% of HRmax. To ensure adherence and accurate exercise progression, all sessions were supervised by exercise physiologist.	The control group continued their habitual lifestyle and habitual physiotherapy treatment.

PP (peak power); MS (multiple sclerosis); VO2max (maximal aerobic capacity); HIIT (high intensity interval training); MCT (moderate continuous training); ACSM (American College of Sports Medicine); DVD (digital versatile disc); 1-RM (1 maximum repetition); PAE (progressive aerobic exercise); HRmax (maximum heart rate).

**Table 5:** Methodological quality of studies.

Study	Downs & Black Scale	PEDro Scale	Levels of Evidence	Grades of Recommendation
Gravesteijn et al. [37]	21/27	8/11	2	B
Ercan et al. [38]	20/27	7/11	2	B
Joisten et al. [39]	20/27	9/11	2	B
Mulero et al. [40]	12/27	NA	4	C
Balaghi et al. [41]	16/27	7/11	2	B
Amiri et al. [42]	12/27	5/11	2	B
Maroto-Izquierdo et al. [43]	14/27	6/11	2	B
Langeskov-Christensen et al. [44]	21/27	7/11	2	B

NA: not applicable.

## Supplementary Materials

The following supporting information can be downloaded at: https://www.mdpi.com/article/10.3390/jcm14030839/s1, Supplementary Materials S1: Downs & Black scale; Supplementary Materials S2: Simulations with Markov Chain Monte Carlo; Supplementary Materials S3: Rankogram.
