# Supplementary material for: Effects of Physical Exercise on Neurofilament Light Chain and Glial Fibrillary Acidic Protein Level in Patients with Multiple Sclerosis: A Systematic Review and Bayesian Network Meta-Analysis
Source: J Clin Med. 2025 Jan 27;14(3):839. doi: 10.3390/jcm14030839 (PMC11818769; doi:10.3390/jcm14030839)
Supplement: Supplementary file 1 [file jcm-14-00839-s001.zip › jcm-3408183-supplementary.pdf]

Supplementary material S1: Downs & Black scale

	Graveste ijn et al[37]	Ercan , et al[38]	Joisen N, et al[39]	Mulero, et al[40]	Balagih et al[41]	Amiri et al 38[42]	Langes kov et el[43]	Maroto et al[44]
**REPORTING**								
1. Is the hypothesis/aim/objective of the study clearly described?	Yes	Yes	Yes	Yes	Yes	Yes	Yes	Yes
2. Are the main outcomes to be measured clearly described in the Introduction or Methods section?	Yes	Yes	Yes	Yes	Yes	Yes	Yes	Yes
3. Are the characteristics of the patients included in the study clearly described?	No	Yes	Yes	Yes	Yes	No	Yes	Yes
4. Are the interventions of interest clearly described?	Yes	Yes	Yes	Yes	Yes	Yes	Yes	Yes
5. Are the distributions of principal confounders in each group of subjects to be compared clearly described?	Yes	No	No	Partially	No	No	No	No
6. Are the main findings of the study clearly described?	Yes	Yes	Yes	Yes	Yes	Yes	Yes	Yes
7. Does the study provide estimates of the random variability in the data for the main outcomes?	Yes	Yes	Yes	No	Yes	Yes	Yes	Yes

8. Have all important adverse events that may be a consequence of the intervention been reported?	No	No	No	No	No	No	No	No
9. Have the characteristics of patients lost to follow-up been described?	Yes	Yes	Yes	Yes	Yes	Yes	Yes	Yes
10. Have actual probability values been reported?	Yes	Yes	Yes	No	Yes	Yes	Yes	Yes
**EXTERNAL VALIDITY**								
11. Were the subjects asked to participate in the study representative of the entire population from which they were recruited?	Yes	Yes	Unable to determi ne	Unable to determi ne	Unable to determi ne	Unable to determi ne	Yes	Unable to determin e
12. Were those subjects who were prepared to participate representative of the entire population from which they were recruited?	Yes	Yes	No	No	No	No	Yes	No
13. Were the staff, places, and facilities where the patients were treated, representative of the treatment the majority of patients receive?	Yes	Yes	Yes	Yes	Yes	No	Yes	No
**INTERNAL VALIDITY - BIAS**								
14. Was an attempt made to blind	No	No	No	No	No	No	No	No

study subjects to the intervention they have received?								
15. Was an attempt made to blind those measuring the main outcomes of the intervention?	Yes	No	Yes	No	No	No	No	No
16. If any of the results of the study were based on "data dredging", was this made clear?	Yes	Yes	Yes	Yes	Yes	Yes	Yes	Yes
17. In trials and cohort studies, do the analyses adjust for different lengths of follow-up of patients, or in case-control studies, is the time period between the intervention and outcome the same for cases and controls?	Yes	Yes	Yes	No	Yes	Unable to determine	Yes	No
18. Were the statistical tests used to assess the main outcomes appropriate?	Yes	Yes	Yes	Yes	Yes	Yes	Yes	Yes
19. Was compliance with the intervention/s reliable?	Yes	Yes	Yes	No	Yes	Yes	Yes	Yes
20. Were the main outcome measures used accurate (valid and reliable)?	Yes	Yes	Yes	Yes	Yes	Yes	Yes	Yes
**INTERNAL VALIDITY - CONFOUNDING**								

21. Were the patients in different intervention groups (trials and cohort studies) or were the cases and controls (case-control studies) recruited from the same population?	Unable to determine	Yes	Yes	No	Unable to determine	Unable to determine	Yes	No
22. Were study subjects in diferent intervention groups (trials and cohort studies) or were the cases and controls (case-control studies) recruited over the same period of time?	Yes	Yes	Yes	No	Unable to determine	Unable to determine	Yes	No
23. Were study subjects randomized to intervention groups?	Yes	No	Yes	No	Unable to determine	Unable to determine	Yes	No
24. Was the randomized intervention assignment concealed from both patients and health care staff until recruitment was complete and irrevocable?	No	No	Yes	No	Unable to determine	Unable to determine	No	No
25. Was there adequate adjustment for confounding in the analyses from which the main findings were drawn?	Yes	No	No	No	No	No	No	No

26. Were losses of patients to follow-up taken into account?	Yes	Yes	Yes	Yes	Yes	Yes	Yes	Yes
**POWER**								
27. Did the study have sufficient power to detect a clinically important effect where the probability value for a difference being due to chance is less than 5%?	No	Yes	No	No	Yes	No	Yes	Yes
**Total Score**	21/27	20/27	20/27	12/27	16/27	12/27	21/27	14/27

Supplementary material S2. Simulations with Markov Chain Monte Carlo.

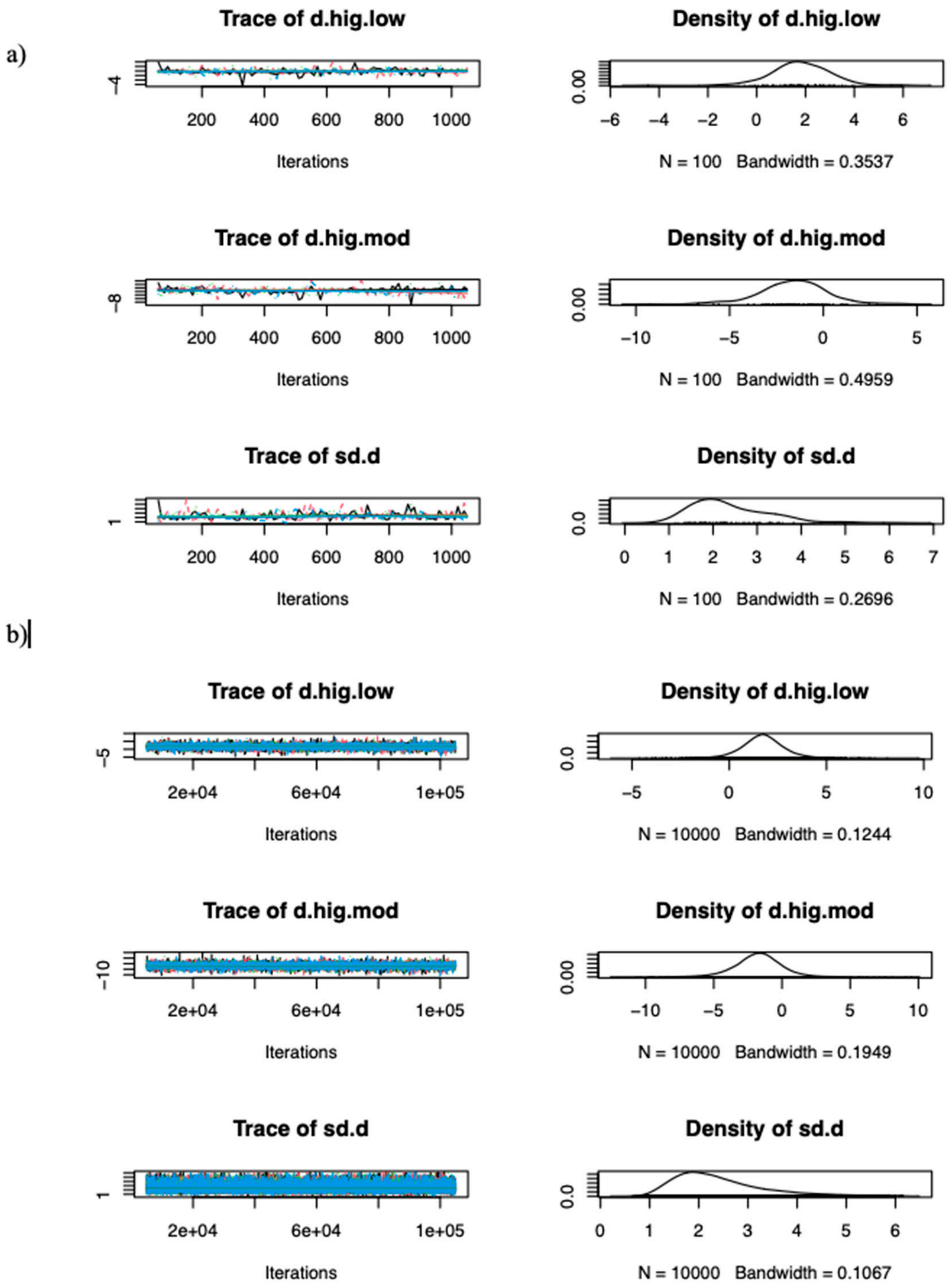

Simulations with Markov Chain Monte Carlo (MCMC) using different numbers of iterations. A) Initial simulation (MCMC1) (Few iterations. B) Extended simulation (MCMC2).

Supplementary Material S3. Rankogram.

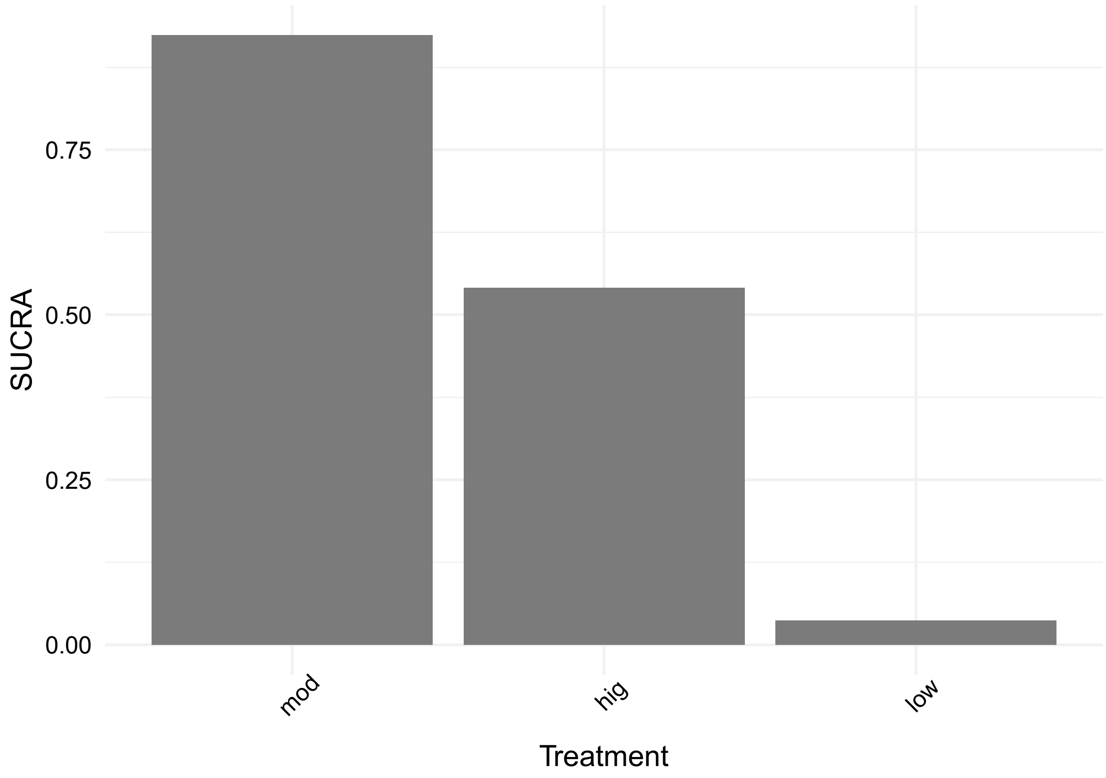

Shows the probability distribution of each treatment occupying different efficacy ranks. It allows visualization of the uncertainty in the rank order of the treatments, where larger areas indicate a higher probability of occupying a specific rank.
